# CancerTrialMatch: a computational resource for the management of biomarker-based clinical trials at a community cancer center

**DOI:** 10.1093/bioinformatics/btaf144

**Published:** 2025-03-31

**Authors:** Padmapriya Swaminathan, Anu Amallraja, Shivani Kapadia, Casey B Williams, Tobias Meißner

**Affiliations:** Department of Cancer Genomics, Avera Cancer Institute, 1000 E 23rd St, Sioux Falls, SD 57105, United States; Department of Cancer Genomics, Avera Cancer Institute, 1000 E 23rd St, Sioux Falls, SD 57105, United States; Department of Epidemiology & Biostatistics, Memorial Sloan Kettering Cancer Center, New York, NY 10065, United States; Department of Cancer Genomics, Avera Cancer Institute, 1000 E 23rd St, Sioux Falls, SD 57105, United States; Department of Hematology & Medical Oncology, Emory University, Atlanta, GA 30322, United States; Department of Cancer Genomics, Avera Cancer Institute, 1000 E 23rd St, Sioux Falls, SD 57105, United States; Department of Cancer Genomics, Avera Cancer Institute, 1000 E 23rd St, Sioux Falls, SD 57105, United States

## Abstract

**Motivation:**

The widespread implementation of next-generation sequencing in cancer care has enabled routine use of molecular and biomarker profiling. At our cancer center, as with many others, biomarker-based clinical trials are increasingly available to oncologists as potential treatment options via molecular tumor boards. To better support this effort, we developed CancerTrialMatch, a systematic approach to capture structured clinical trial data and match patients to trials based on their disease characteristics and sequencing profiles.

**Results:**

CancerTrialMatch is an open-source application designed to streamline clinical trial curation and patient trial matching, while also enabling an institution’s curated trial portfolio to be distributed across the institution for easy access to providers, care teams and researchers. It facilitates curating, updating, and searching for trials through a semi-automated interface built using R Shiny, MongoDB, and Docker. While much of the trial data is retrieved via the clinicaltrials.gov Application Programming Interface, certain items like biomarkers and disease subtypes are entered manually. The user inputs disease type using the OncoTree classification, and provides relevant biomarker details, such as mutations, copy numbers, fusions, and other disease-specific markers. This resource reduces the time required for institutional trial management and helps to identify potential clinical trials for patients, ultimately supporting larger clinical trial enrollment and enhancing the clinical application of precision oncology.

**Availability and implementation:**

CancerTrialMatch was implemented and tested on Windows 11 (64-bit, 32 GB RAM) using WSL2 with Ubuntu 22.04. Docker 27.0.3 and Docker Compose 2.28.1 were used to build images and containers. Users can build it by cloning the repo and following the README instructions and supplemental file (cancertrialmatchsupplemental.pdf) . The source code and example data are available in GitHub and Figshare at https://github.com/AveraSD/CancerTrialMatch and 10.6084/m9.figshare.28447367 respectively.

## 1 Introduction

Next-generation sequencing has made comprehensive genomic profiling more accessible to cancer patients, thus allowing for more targeted treatment options. At our institution, various clinical trials including cooperative group studies, industry sponsored studies, investigator-initiated studies, and just-in-time trials are available, providing opportunities for patient enrollment based on disease and molecular profiles. Patients are matched to appropriate trials after thorough review at disease-specific or molecular tumor boards. As the number of available trials grows, managing and tracking the vast amount of information becomes increasingly complex. It is crucial that this information is organized and readily accessible to clinicians in a concise and user-friendly manner.

A search for solutions to this challenge showed only one viable option, MatchMiner (Klein *et al.* 2022, Nierengarten 2023). While it was a comprehensive application, the extensive and complex setup was out of scope for a smaller institution such as ours. Due to these challenges, we developed CancerTrialMatch, an open-source application that is lightweight and containerized. Our application is designed to accommodate both biomarker and non-biomarker-based clinical trials with customizations that prioritize relevant trials. This enables clinicians to easily browse all available trials, thereby simplifying trial identification.

## 2 Methods

**Figure 1.**
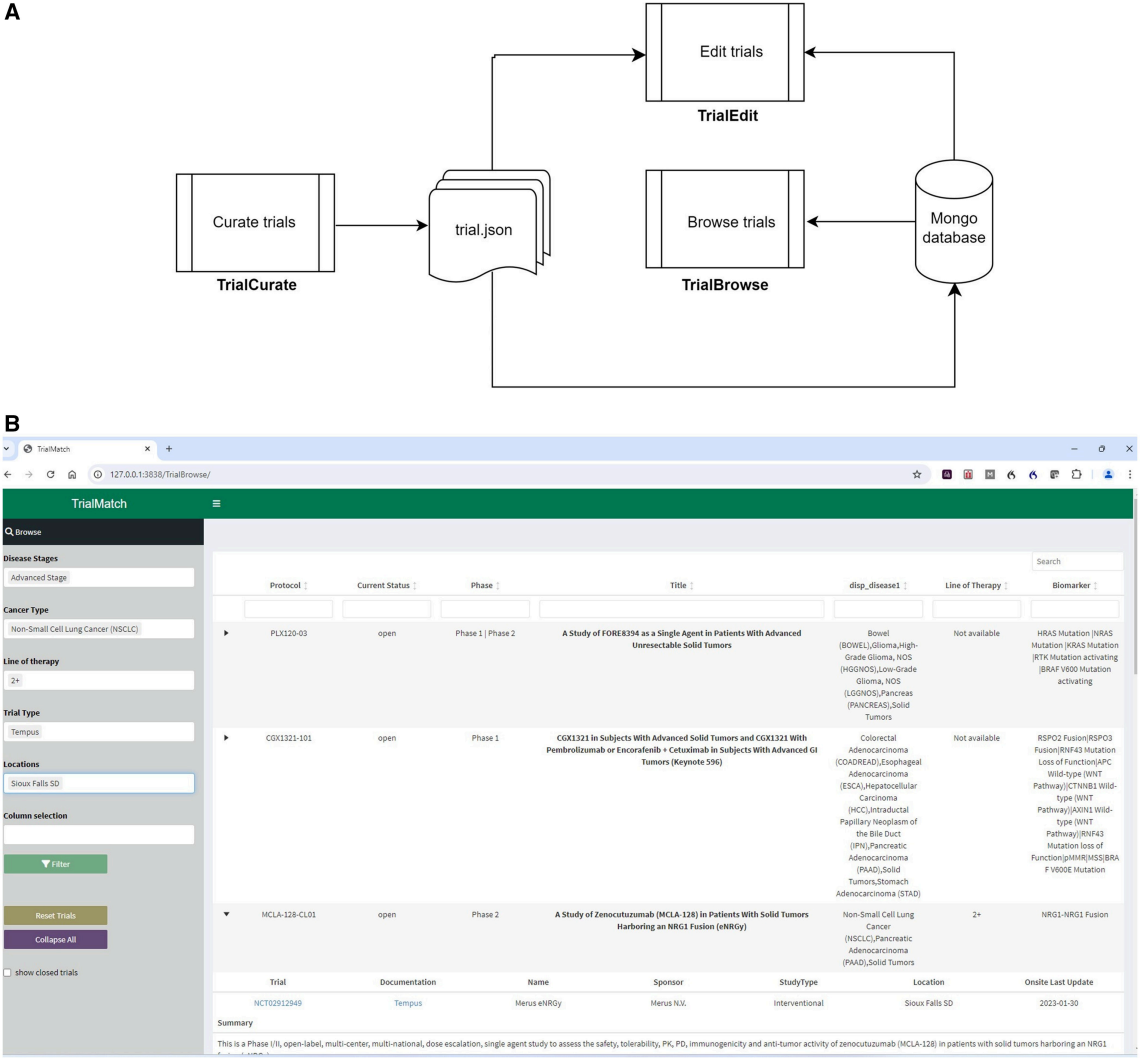
CancerTrialMatch: (A) flowchart showing the relationship between the three apps. (B) Screenshot of TrialBrowse application with results after search.

CancerTrialMatch, developed using R Shiny, provides three shiny apps: (i) TrialCurate: a semi-automated shiny app for adding clinical trials, (ii) TrialEdit: a shiny app to edit existing clinical trials, and (iii) TrialBrowse: a shiny app for browsing and searching clinical trials. The backend of the application uses MongoDB to store clinical trial data, and Docker is used to manage software installation and application deployment (Fig. 1).

- *TrialCurate* app allows users to input key details such as trial name, NCT identifier from clinicaltrials.gov, trial status, and classification. This information is used to query the clinicaltrials.gov Application Programming Interface (API), retrieving data like the title, summary statement, trial phase, sponsor, and more. However, details such as disease stage, biomarkers and inclusion/exclusion criteria are not available via the API as discrete fields, requiring manual input. The user can classify disease types using the OncoTree (Kundra *et al.* 2021) oncology classification system, enter biomarker information including mutations, copy number alterations, fusions, tumor mutational burden, microsatellite instability, PD-L1, gene expression and disease specific markers like ER/PR/HER2 (for breast cancer). Additionally, users provide information about trial cohort arm status and line of therapy. Each trial’s treatment protocol is made accessible via a hyperlink, along with the date of its most recent update. Once this trial information is curated, it is reviewed and saved as a JavaScript Object Notation (JSON) document to a MongoDB collection.
- *TrialEdit* app allows users to edit any previously curated clinical trial by retrieving the relevant information from the MongoDB. On the left panel, a list of clinical trials and their respective identifiers is displayed, along with the protocol details. Once a clinical trial is selected, its corresponding information is displayed on the right organized in two sections. Each section contains its own save button for specific changes, and a final save button is available to store the edited trial back into the MongoDB.
- *TrialBrowse* app serves as the primary user interface, allowing users to browse all trials stored in the MongoDB. It includes a filter panel for refining search results based on multiple criteria. Once filtered, the matching trials are displayed in a results table on the right-hand side of the filter panel. Each trial is linked to its clinicaltrials.gov identifier, displayed as a hyperlink for additional details. Users can also expand each trial entry to view more comprehensive information.

## 3 Conclusions

CancerTrialMatch is designed to support clinicians, staff, and researchers in identifying and recommending potential clinical trial options to cancer patients. By enabling detailed curation, precise editing, and efficient browsing of an institution’s clinical trial portfolio, this resource enables the application of precision medicine. With the MongoDB backend, it offers scalability to accommodate a growing number of biomarker-based clinical trials.

These features streamline the process of identifying suitable trials, enhance clinical trial enrollment, and contribute to personalized cancer care. By leveraging data management and curation capabilities, CancerTrialMatch ensures that clinicians can make informed decisions, ultimately improving patient outcomes.

## Supplementary Material

btaf144_Supplementary_Data

## References

[btaf144-B1] Klein H , Mazor T, Siegel E et al MatchMiner: an open-source platform for cancer precision medicine. NPJ Precis Oncol 2022;6:69. 36202909 10.1038/s41698-022-00312-5 PMC9537311

[btaf144-B2] Kundra R , Zhang H, Sheridan R et al OncoTree: a cancer classification system for precision oncology. ICO Clin Cancer Inform 2021;5:221–30. 10.1200/CCI.20.00108 PMC8240791 33625877

[btaf144-B3] Nierengarten MB. MatchMiner open-source platform matches patients with cancer to precision medicine trials. Cancer 2023;129:494. 36651152 10.1002/cncr.34649

