# Supplementary material for: CancerTrialMatch: a computational resource for the management of biomarker-based clinical trials at a community cancer center
Source: Bioinformatics. 2025 Mar 31;41(4):btaf144. doi: 10.1093/bioinformatics/btaf144 (PMC12002907; doi:10.1093/bioinformatics/btaf144)
Supplement: btaf144_Supplementary_Data [file btaf144_supplementary_data.pdf]

# CancerTrialMatch

TrialCurate, TrialEdit, TrialBrowse are the three shiny apps to curate, edit and search clinical trials.

The source code for the three shiny apps: TrialCurate, TrialEdit, TrialBrowse are available at

https://github.com/AveraSD/CancerTrialMatch/TrialCurate

https://github.com/AveraSD/CancerTrialMatch/TrialEdit

https://github.com/AveraSD/CancerTrialMatch/TrialBrowse

Examples of NCT identifiers: NCT02428712, NCT03662126, NCT05361395 to use for curation.

Install Docker desktop and enable WSL integration in the case of windows.

All the three shiny apps are dockerized and the instructions to build and test docker images using the command line are as follows:

**Go to the main folder where Dockerfile is located and do:**

docker build -t shiny-apps .

**Go to the folder where docker-compose is located and do:**

docker-compose up --build

**To check docker images:**

docker ps

**To check docker containers:**

docker ps -a

**Access the Shiny apps through a web browser at:**

http://127.0.0.1/TrialCurate/

http://127.0.0.1/TrialEdit/

http://127.0.0.1/TrialBrowse/

**To remove and clean up docker images, containers, builds etc.**

docker-compose down -v

**To curate a clinical trial:**

**Connect to clinicaltrials.gov API**

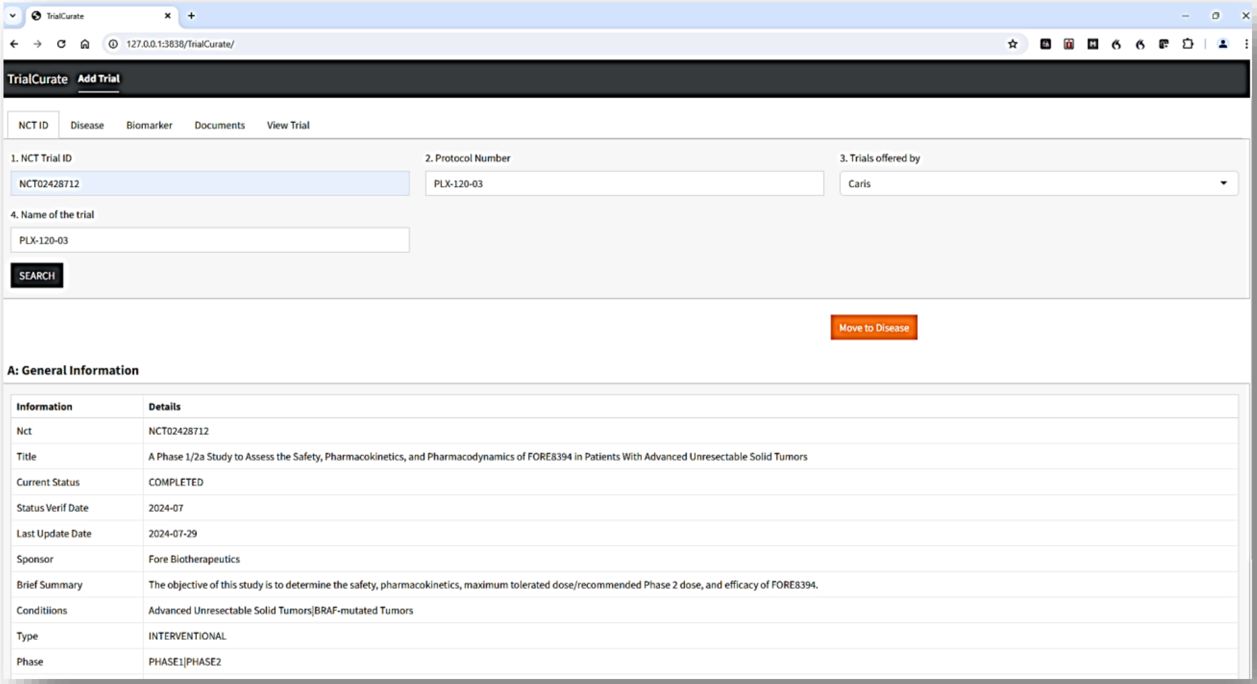

## Disease and stage

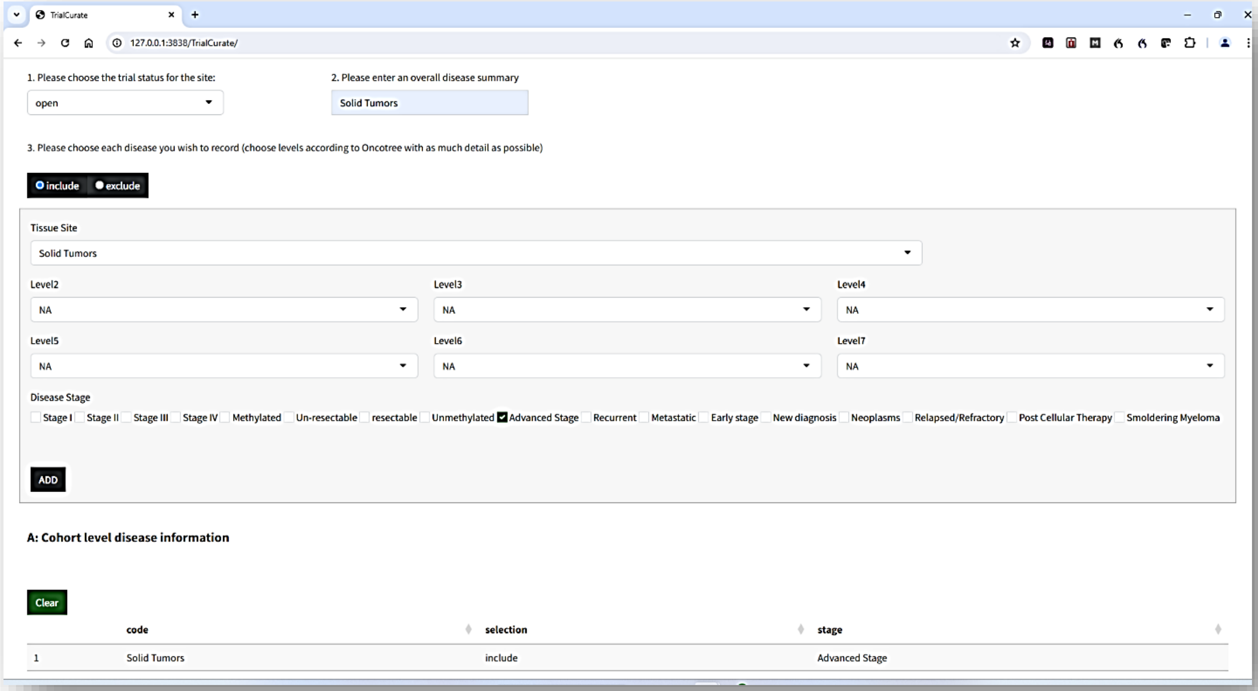


## Line of therapy and arm status

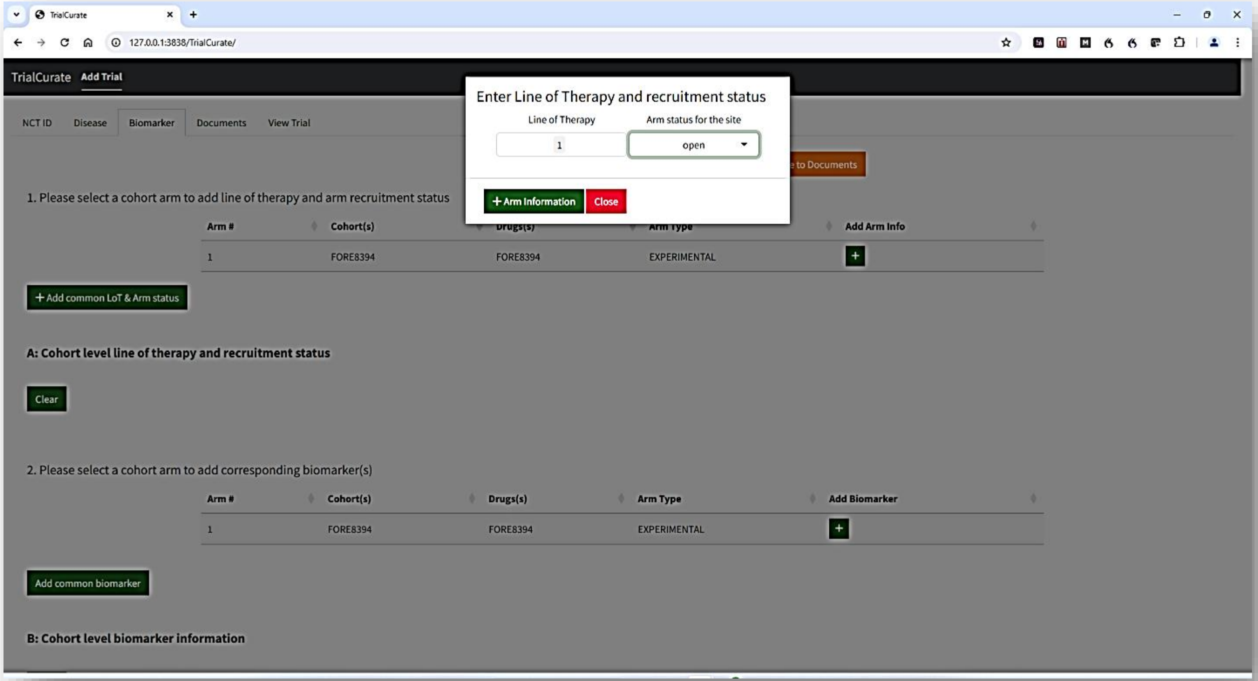

## Enter biomarker information

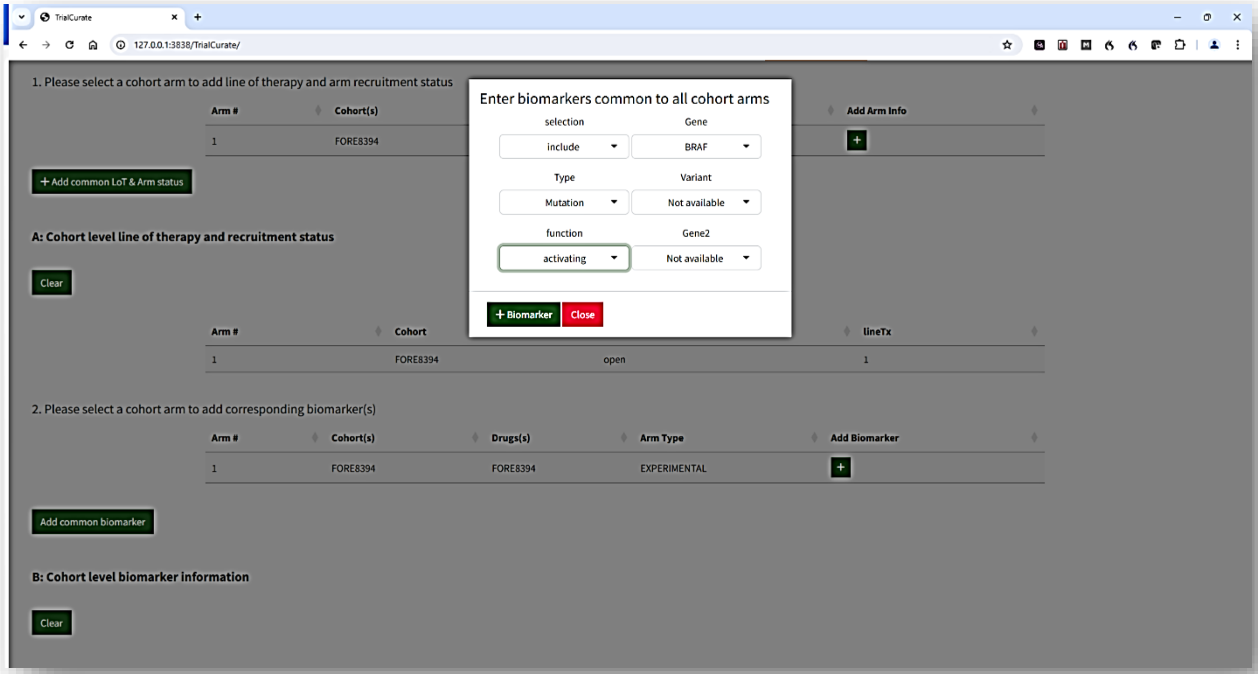


## Biomarker details overview

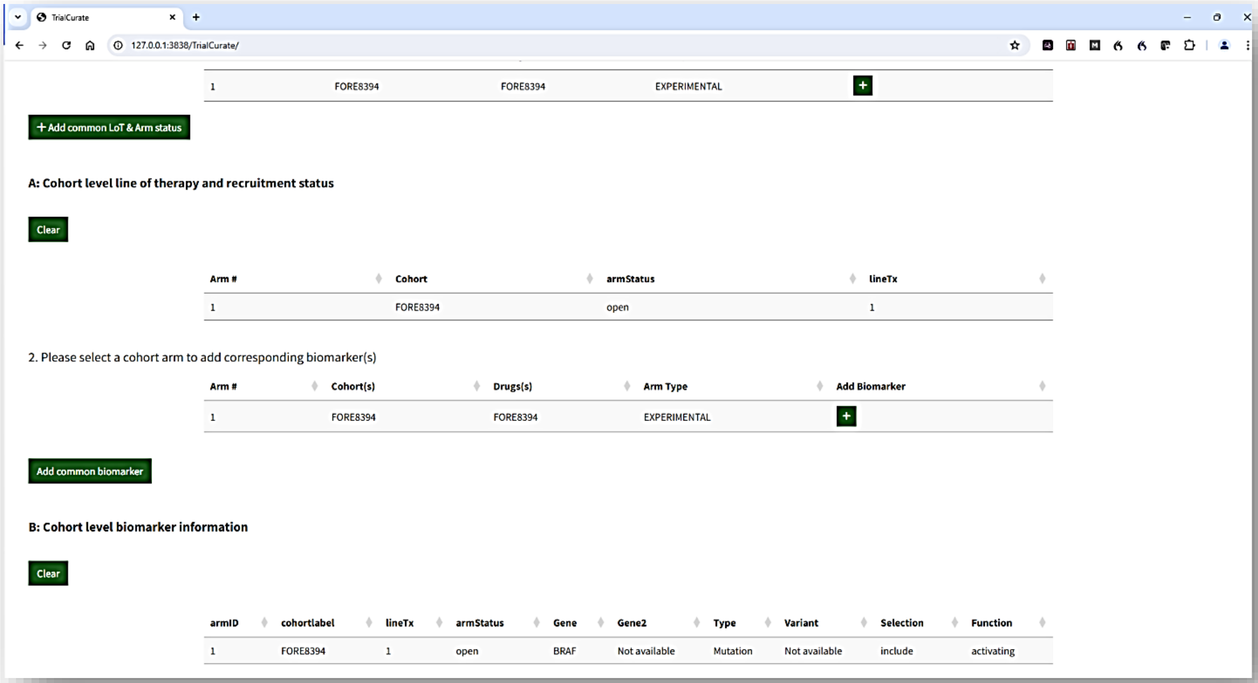

## Documentation

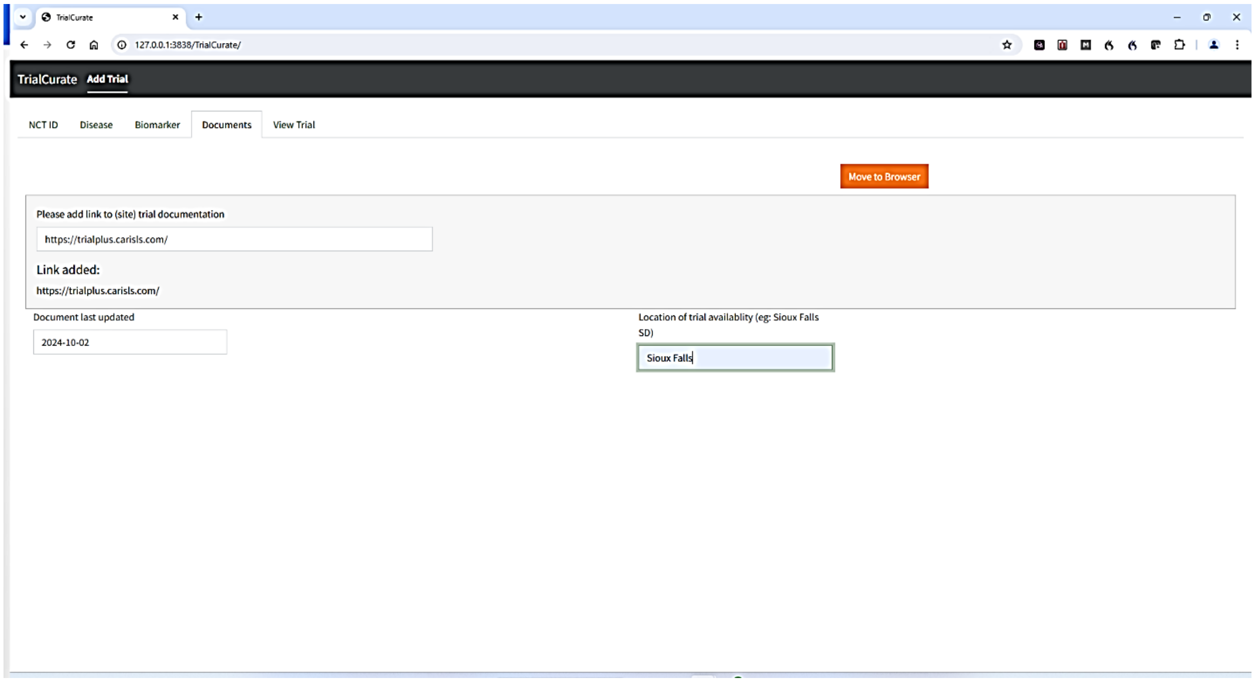


## Overview and confirm

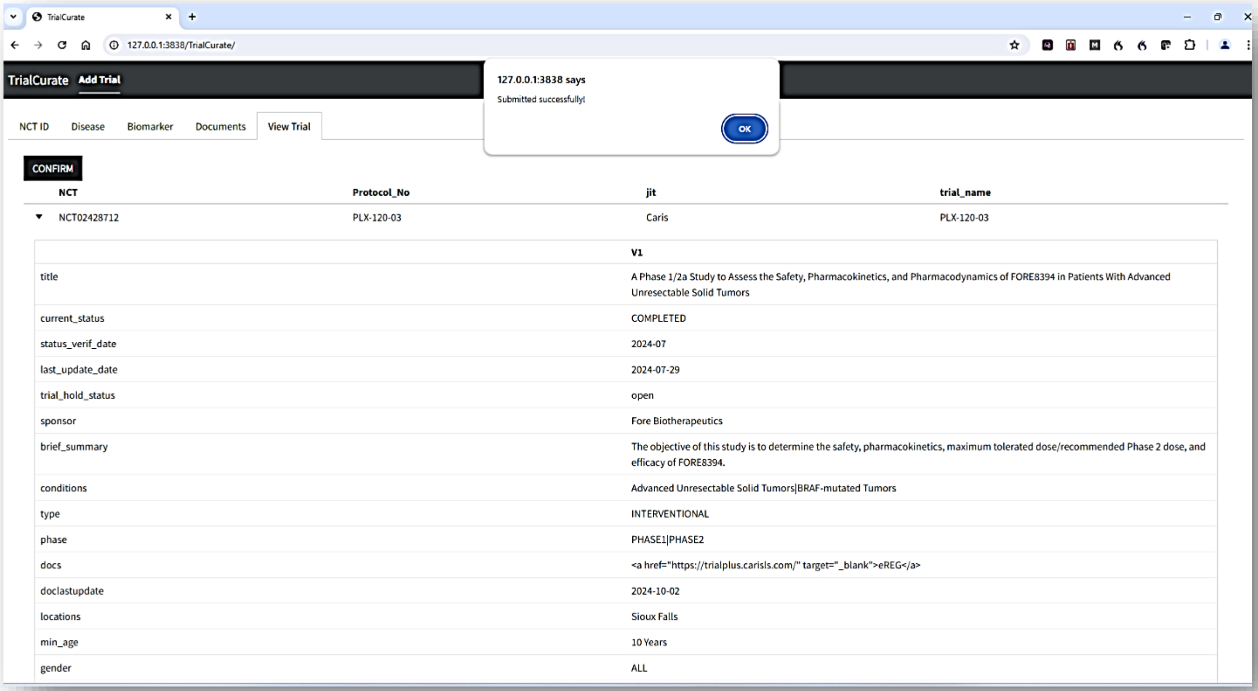

**Check curation results**

To check if the clinical trial you curated is saved correctly and exists in mongo database, check by doing

docker exec -it mongocontainerid mongosh

This takes you inside the mongo database interface. Then type the following commands at the prompt:

(aci is database name and ClinicalTrials is collection name)

use aci

db.ClinicalTrials.countDocuments()
You should see 1

db.ClinicalTrials.find({“info.NCT”: “yourNCTid”})

This should show the complete information for the curated clinical trial

**To edit a clinical trial:**

**How to edit existing trials using TrialEdit interface**

**Select trial to edit**

**There are two parts: Trial Part I (consists of 4 sections) and Trial Part 2 (consists of 2 sections) and a submit button on the top of the page to finally submit the trial after making changes.**

**Each section has a table and click on any cell inside the table where you need to make a change and then click on "Save Verify" button to save the change you made.**

**In case you do not have any change in any section, you still must click on "Save Verify" button in each section.**

**Click on the "Save Verify" button on all sections and then hit the "Submit button" on the top of the page to save the trial to the database. This will avoid the "Disconnected from the server" error.**

**Select trial to edit**

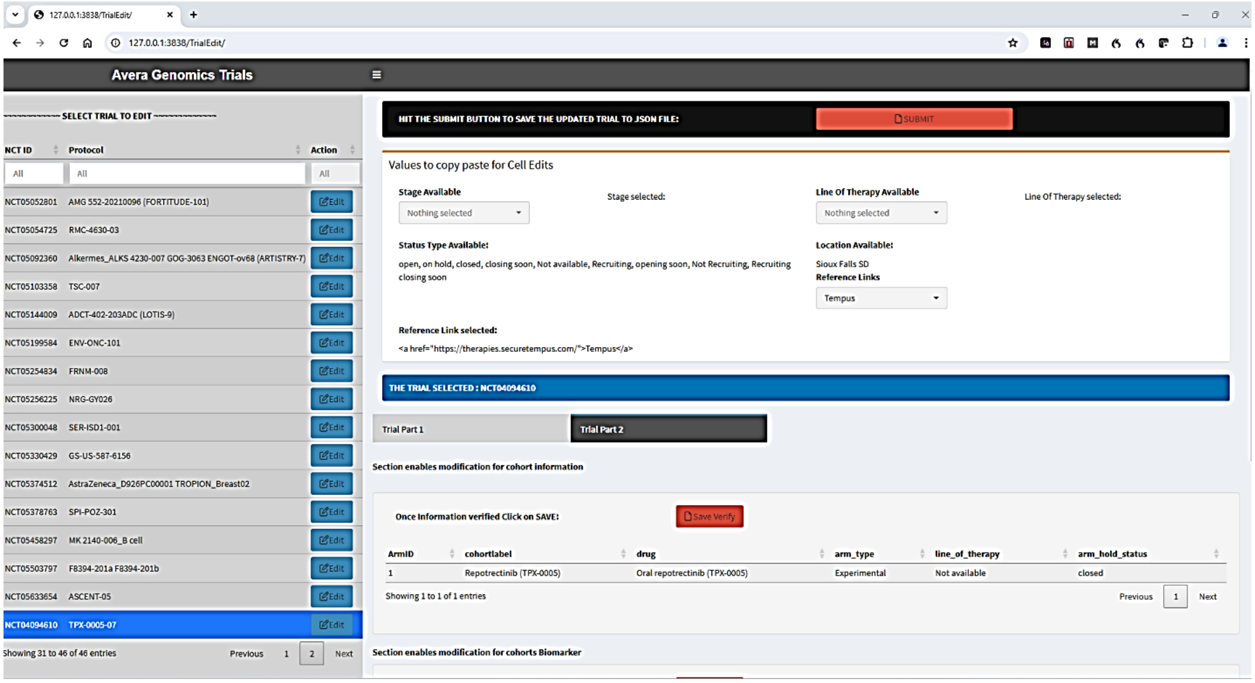


**Trial Part I**

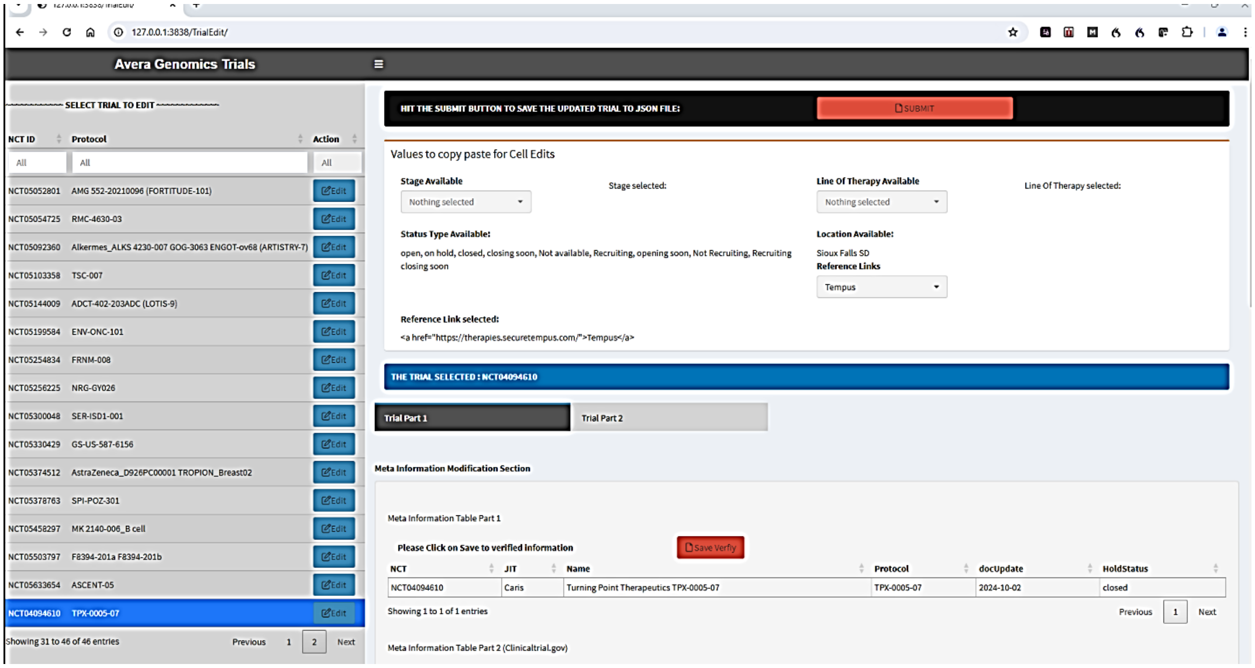


# Trial Part I

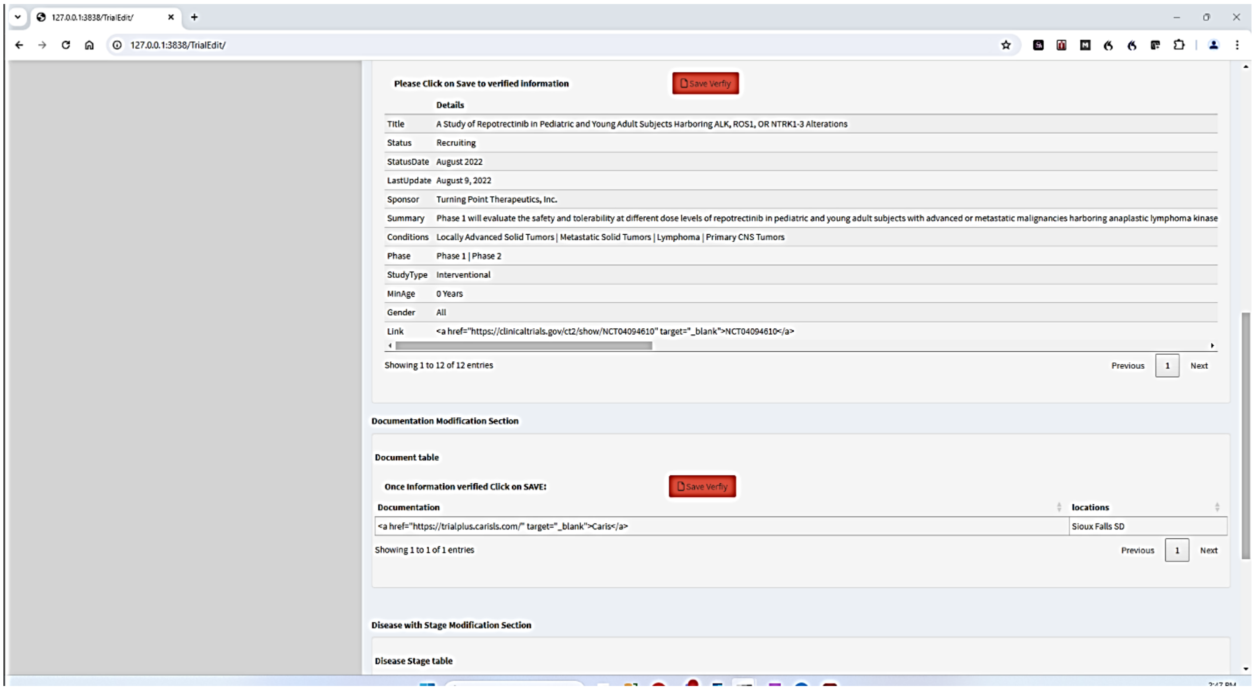


# Trial Part I

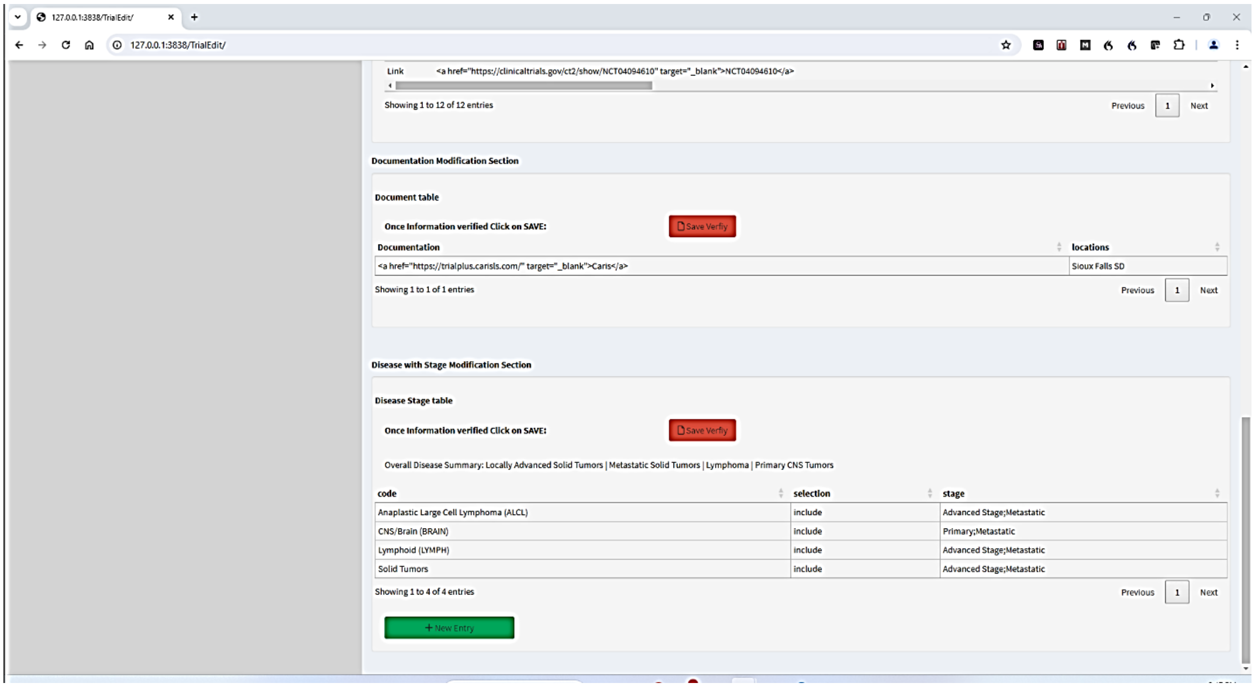


## Trial Part II

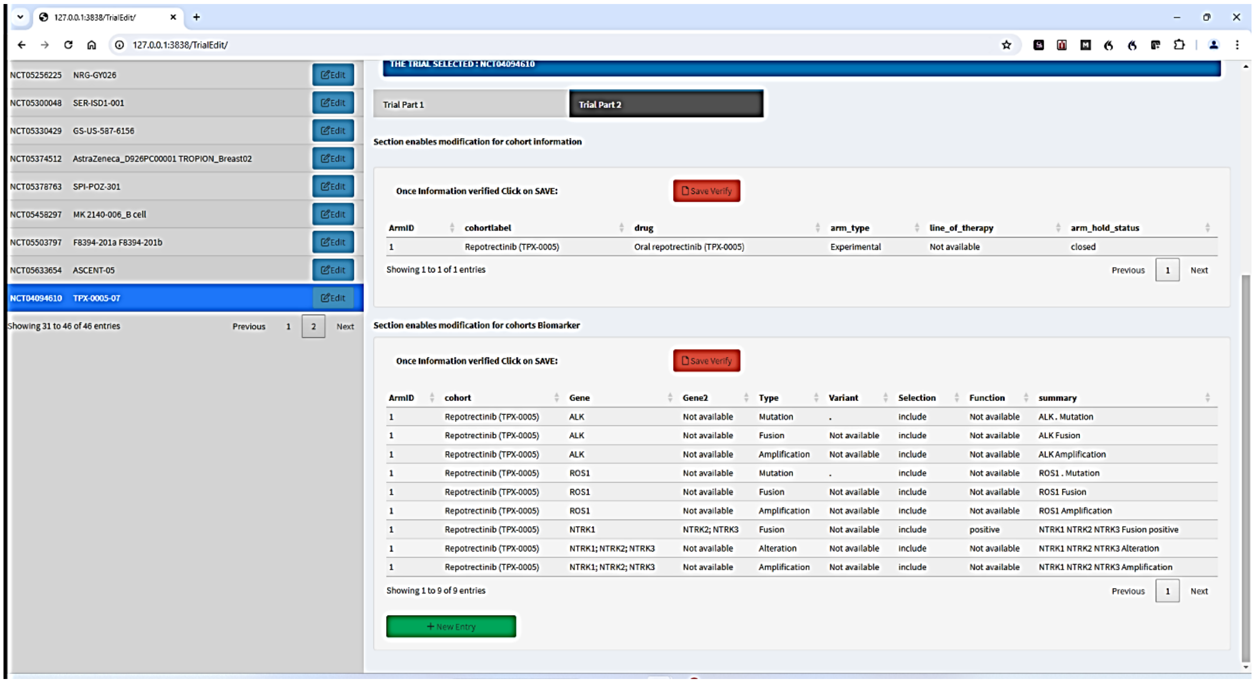

**Check edited trial:**

- Repeat the same steps as you did above to check the newly curated trial. You will see the change you saved for the selected clinical trial in the mongo database.
- You can also check directly in the TrialEdit interface by clicking the trial and looking at the two tabs Trial Part 1 and Trial Part 2.

**To browse clinical trials:**

**Browse main display**

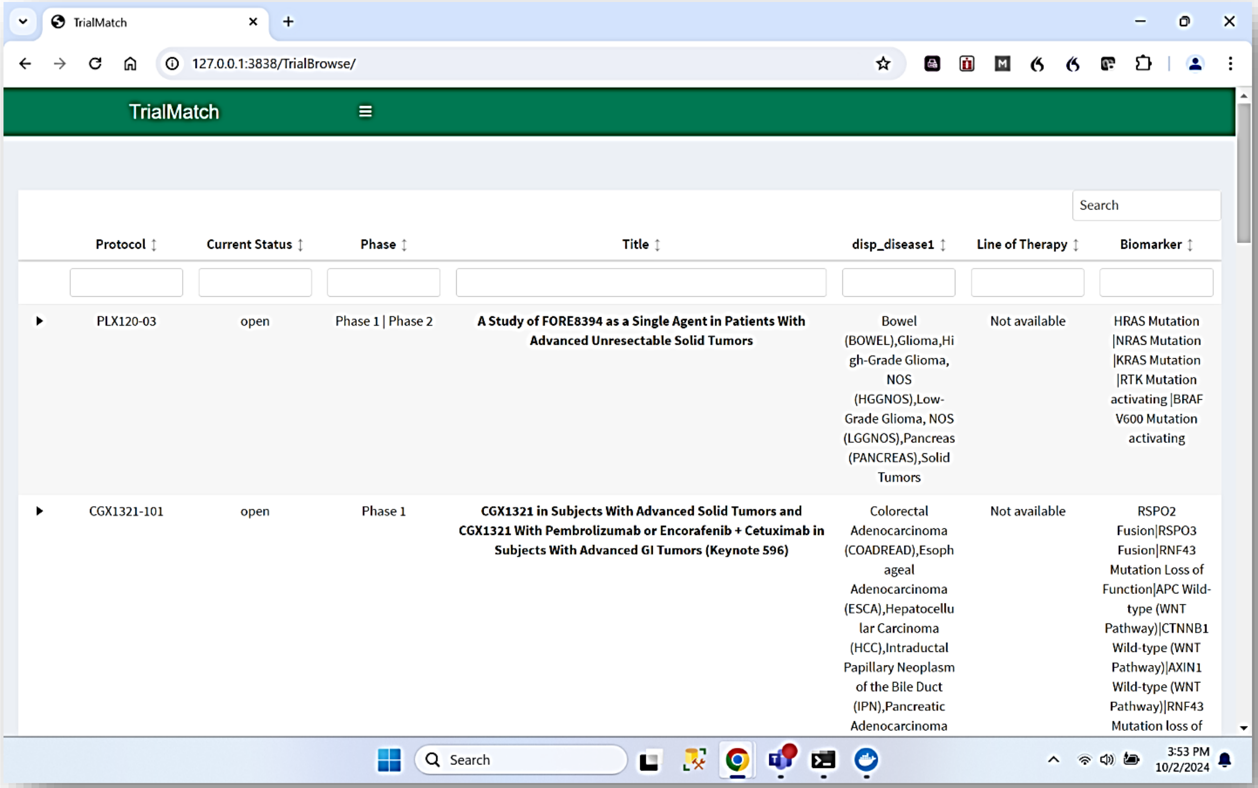

## Browse with sidebar menu

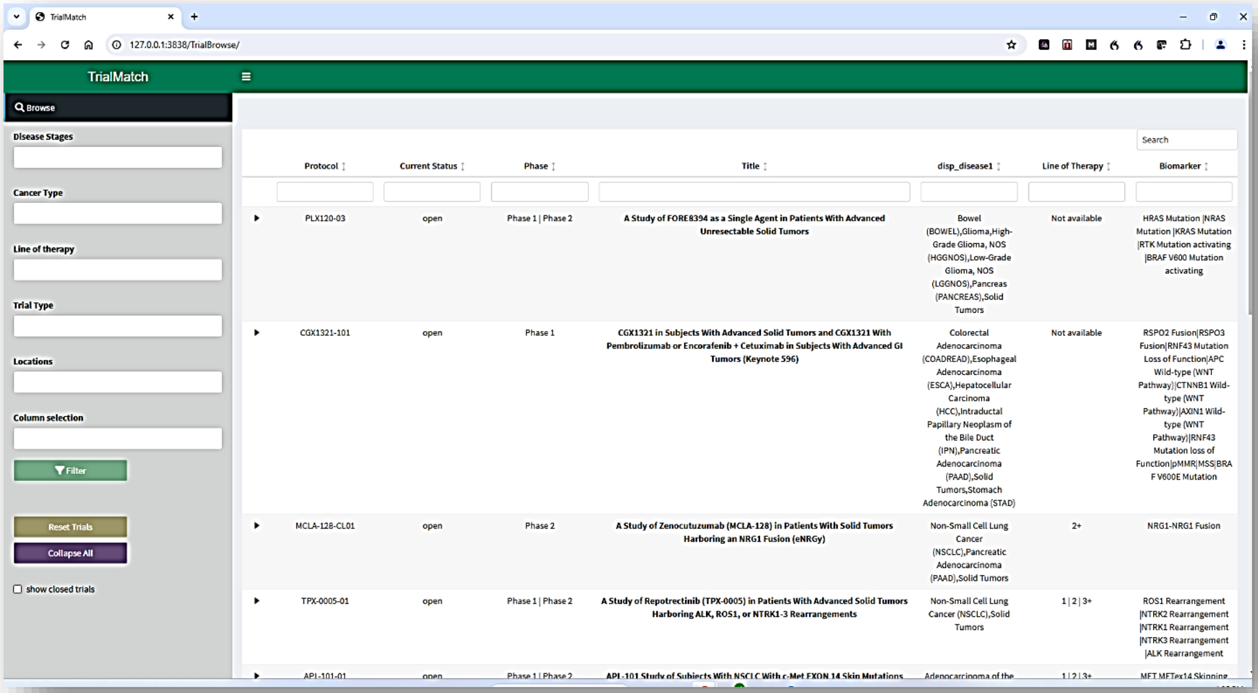


## Browse with closed clinical trials

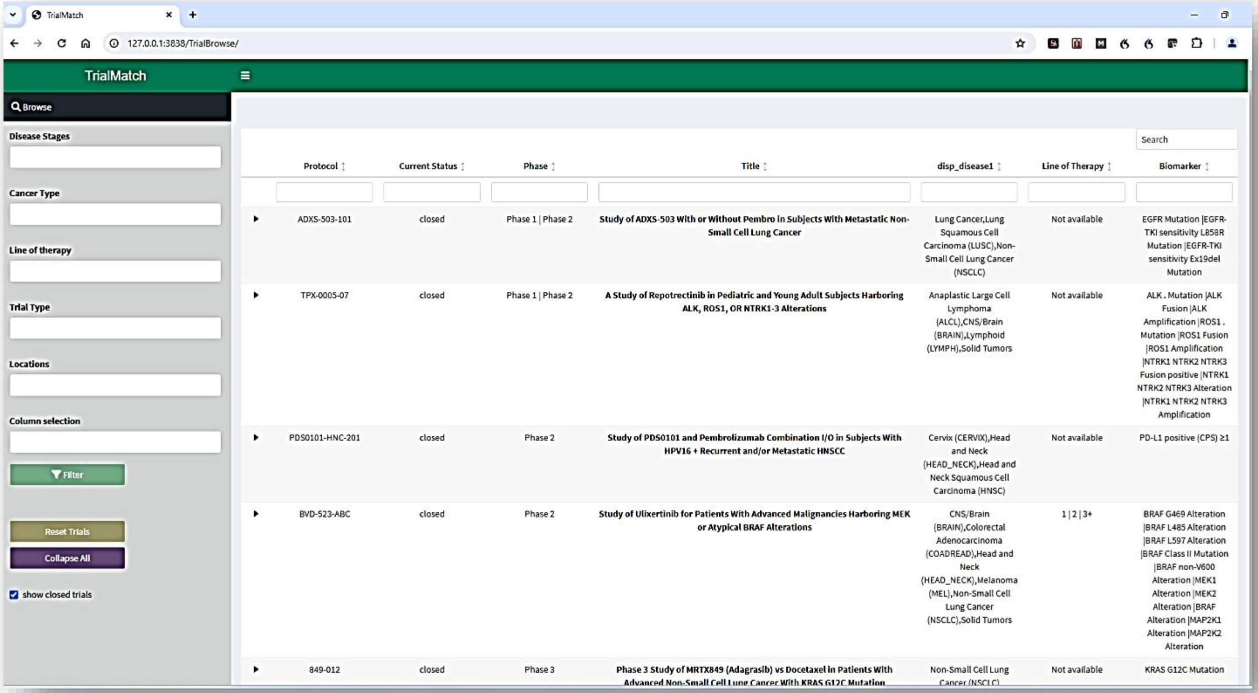

## Browse with search criteria

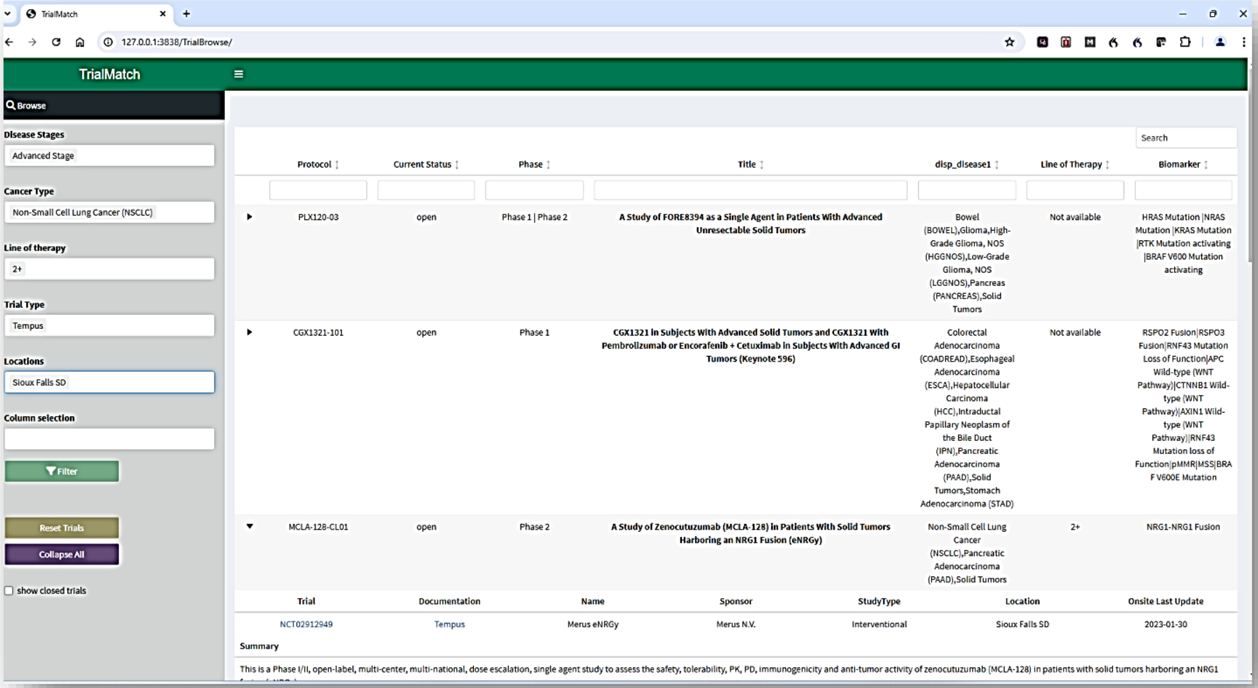


## Browse with column selection

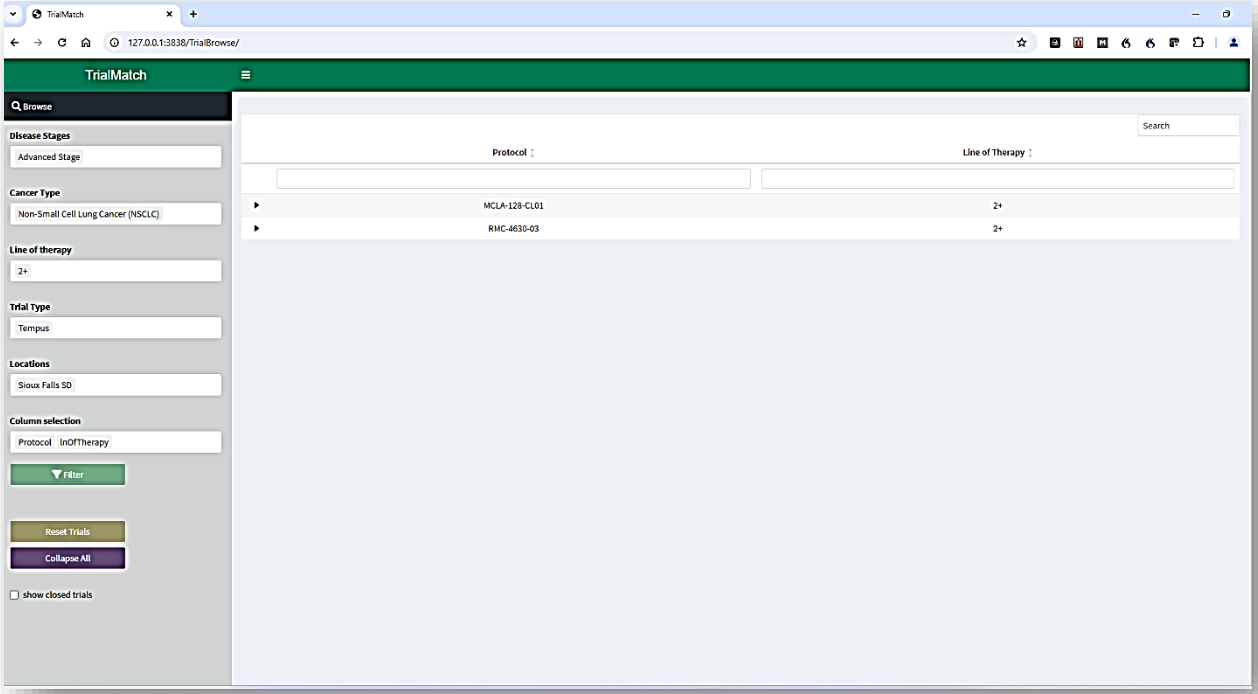

**Browse with search box**

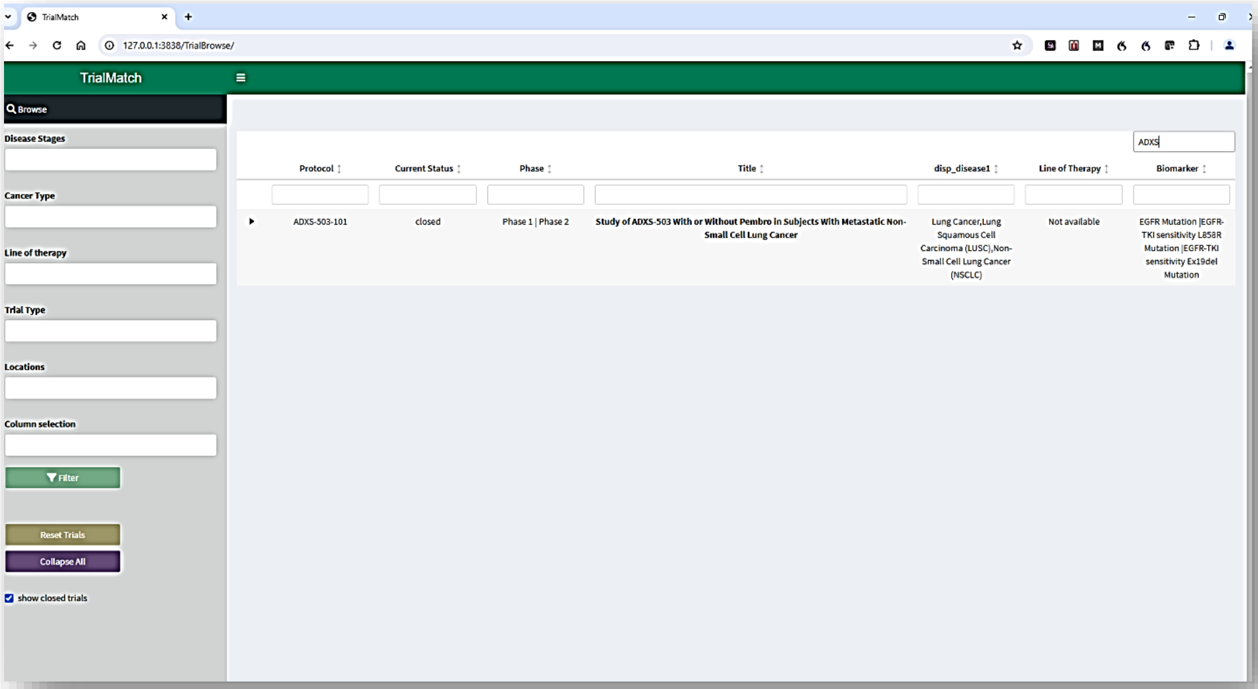


**Check clinical trials using TrialBrowse interface**

This app when invoked will load a few trials from the database.

- To check just load the page using the URL.
- Once it is loaded, click on the hamburger icon and this opens the sidebar and shows the search menu. Enter values for the different search criteria and filter will show the trials in the table on the right. Click on Reset trials clear the results.
- You can expand information to read more details for each trial and look at the closed trials by clicking on show closed trials checkbox.
- Search bar on the top right helps to search the full table with a keyword.

Please see below for more information about R packages and their versions.

R version 4.2.2 (2022-10-31) -- "Innocent and Trusting"
Copyright (C) 2022 The R Foundation for Statistical Computing
Platform: x86_64-pc-linux-gnu (64-bit)

```
> installed.packages()[,c('Package', 'Version')]
           Package       Version
anytime    "anytime"     "0.3.9"
AsioHeaders "AsioHeaders" "1.22.1-2"
askpass    "askpass"     "1.2.0"
backports  "backports"   "1.5.0"
base64enc  "base64enc"   "0.1-3"
bench      "bench"       "1.1.3"
BH         "BH"          "1.84.0-0"
bit        "bit"         "4.0.5"
bit64      "bit64"       "4.0.5"
blob       "blob"        "1.2.4"
brio       "brio"        "1.1.5"
broom      "broom"       "1.0.6"
bsicons    "bsicons"     "0.1.2"
bslib      "bslib"       "0.8.0"
cachem     "cachem"      "1.1.0"
callr      "callr"       "3.7.6"
cellranger "cellranger"  "1.1.0"
checkmate  "checkmate"   "2.3.2"
classInt   "classInt"    "0.4-10"
cli        "cli"         "3.6.3"
clipr      "clipr"       "0.8.0"
colorspace "colorspace"  "2.1-1"
commonmark "commonmark"  "1.9.1"
config     "config"      "0.3.2"
conflicted "conflicted"  "1.2.0"
covr       "covr"        "3.6.4"
cpp11      "cpp11"       "0.5.0"
crayon     "crayon"      "1.5.3"
crosstalk  "crosstalk"   "1.2.1"
curl       "curl"        "5.2.2"
data.table "data.table"  "1.16.0"
DBI        "DBI"         "1.2.3"
dbplyr     "dbplyr"      "2.5.0"
```

| | | |
|---|---|---|
| desc | "desc" | "1.4.3" |
| diffobj | "diffobj" | "0.3.5" |
| digest | "digest" | "0.6.37" |
| docopt | "docopt" | "0.7.1" |
| dplyr | "dplyr" | "1.1.4" |
| DT | "DT" | "0.33" |
| dtplyr | "dtplyr" | "1.3.1" |
| dygraphs | "dygraphs" | "1.1.1.6" |
| e1071 | "e1071" | "1.7-14" |
| evaluate | "evaluate" | "0.24.0" |
| fansi | "fansi" | "1.0.6" |
| farver | "farver" | "2.1.2" |
| fastmap | "fastmap" | "1.2.0" |
| feather | "feather" | "0.3.5" |
| fontawesome | "fontawesome" | "0.5.2" |
| forcats | "forcats" | "1.0.0" |
| fresh | "fresh" | "0.2.1" |
| fs | "fs" | "1.6.4" |
| future | "future" | "1.34.0" |
| gargle | "gargle" | "1.5.2" |
| generics | "generics" | "0.1.3" |
| ggplot2 | "ggplot2" | "3.5.1" |
| globals | "globals" | "0.16.3" |
| glue | "glue" | "1.7.0" |
| googledrive | "googledrive" | "2.1.1" |
| googlesheets4 | "googlesheets4" | "1.1.1" |
| gtable | "gtable" | "0.3.5" |
| haven | "haven" | "2.5.4" |
| here | "here" | "1.0.1" |
| highr | "highr" | "0.11" |
| hms | "hms" | "1.1.3" |
| htmltools | "htmltools" | "0.5.8.1" |
| htmlwidgets | "htmlwidgets" | "1.6.4" |
| httpuv | "httpuv" | "1.6.15" |
| httr | "httr" | "1.4.7" |
| hunspell | "hunspell" | "3.0.4" |
| ids | "ids" | "1.0.1" |
| isoband | "isoband" | "0.2.7" |
| jpeg | "jpeg" | "0.1-10" |
| jquerylib | "jquerylib" | "0.1.4" |
| jsonlite | "jsonlite" | "1.8.8" |

| | | |
|---|---|---|
| knitr | "knitr" | "1.48" |
| labeling | "labeling" | "0.4.3" |
| Lahman | "Lahman" | "11.0-0" |
| later | "later" | "1.3.2" |
| lazyeval | "lazyeval" | "0.2.2" |
| leaflet.providers | "leaflet.providers" | "2.0.0" |
| lifecycle | "lifecycle" | "1.0.4" |
| listenv | "listenv" | "0.9.1" |
| littler | "littler" | "0.3.17" |
| lobstr | "lobstr" | "1.1.2" |
| lubridate | "lubridate" | "1.9.3" |
| magrittr | "magrittr" | "2.0.3" |
| markdown | "markdown" | "1.13" |
| memoise | "memoise" | "2.0.1" |
| microbenchmark | "microbenchmark" | "1.5.0" |
| mime | "mime" | "0.12" |
| mockr | "mockr" | "0.2.1" |
| modelr | "modelr" | "0.1.11" |
| mongolite | "mongolite" | "2.8.0" |
| munsell | "munsell" | "0.5.1" |
| nycflights13 | "nycflights13" | "1.0.2" |
| openssl | "openssl" | "2.2.1" |
| palmerpenguins | "palmerpenguins" | "0.1.1" |
| parallelly | "parallelly" | "1.38.0" |
| pillar | "pillar" | "1.9.0" |
| pkgbuild | "pkgbuild" | "1.4.4" |
| pkgconfig | "pkgconfig" | "2.0.3" |
| pkgload | "pkgload" | "1.4.0" |
| plogr | "plogr" | "0.2.0" |
| plyr | "plyr" | "1.8.9" |
| png | "png" | "0.1-8" |
| praise | "praise" | "1.0.0" |
| prettyunits | "prettyunits" | "1.2.0" |
| processx | "processx" | "3.8.4" |
| profmem | "profmem" | "0.6.0" |
| progress | "progress" | "1.2.3" |
| promises | "promises" | "1.3.0" |
| proxy | "proxy" | "0.4-27" |
| ps | "ps" | "1.7.7" |
| purrr | "purrr" | "1.0.2" |
| R.cache | "R.cache" | "0.16.0" |

| | | |
|---|---|---|
| R.methodsS3 | "R.methodsS3" | "1.8.2" |
| R.oo | "R.oo" | "1.26.0" |
| R.rsp | "R.rsp" | "0.46.0" |
| R.utils | "R.utils" | "2.12.3" |
| R6 | "R6" | "2.5.1" |
| ragg | "ragg" | "1.3.2" |
| rappdirs | "rappdirs" | "0.3.3" |
| RColorBrewer | "RColorBrewer" | "1.1-3" |
| Rcpp | "Rcpp" | "1.0.13" |
| reactable | "reactable" | "0.4.4" |
| reactablefmtr | "reactablefmtr" | "2.0.0" |
| reactlog | "reactlog" | "1.1.1" |
| reactR | "reactR" | "0.6.0" |
| readr | "readr" | "2.1.5" |
| readxl | "readxl" | "1.4.3" |
| rematch | "rematch" | "2.0.0" |
| rematch2 | "rematch2" | "2.1.2" |
| reprex | "reprex" | "2.1.1" |
| repurrrsive | "repurrrsive" | "1.1.0" |
| rex | "rex" | "1.2.1" |
| rlang | "rlang" | "1.1.4" |
| rmarkdown | "rmarkdown" | "2.28" |
| rprojroot | "rprojroot" | "2.0.4" |
| RSQLite | "RSQLite" | "2.3.7" |
| rstudioapi | "rstudioapi" | "0.16.0" |
| rvest | "rvest" | "1.0.4" |
| s2 | "s2" | "1.1.7" |
| sass | "sass" | "0.4.9" |
| scales | "scales" | "1.3.0" |
| selectr | "selectr" | "0.4-2" |
| shiny | "shiny" | "1.9.1" |
| shinyAce | "shinyAce" | "0.4.2" |
| shinydashboard | "shinydashboard" | "0.7.2" |
| shinydashboardPlus | "shinydashboardPlus" | "2.0.5" |
| shinydisconnect | "shinydisconnect" | "0.1.1" |
| shinyFiles | "shinyFiles" | "0.9.3" |
| shinyjs | "shinyjs" | "2.1.0" |
| shinythemes | "shinythemes" | "1.2.0" |
| shinyWidgets | "shinyWidgets" | "0.8.6" |
| showtext | "showtext" | "0.9-7" |
| showtextdb | "showtextdb" | "3.0" |

| | | |
|---|---|---|
| sourcetools | "sourcetools" | "0.1.7-1" |
| sp | "sp" | "2.1-4" |
| sparkline | "sparkline" | "2.0" |
| spelling | "spelling" | "2.3.0" |
| stringi | "stringi" | "1.8.4" |
| stringr | "stringr" | "1.5.1" |
| sys | "sys" | "3.4.2" |
| sysfonts | "sysfonts" | "0.8.9" |
| systemfonts | "systemfonts" | "1.1.0" |
| testit | "testit" | "0.13" |
| testthat | "testthat" | "3.2.1.1" |
| textshaping | "textshaping" | "0.4.0" |
| thematic | "thematic" | "0.1.6" |
| tibble | "tibble" | "3.2.1" |
| tidyr | "tidyr" | "1.3.1" |
| tidyselect | "tidyselect" | "1.2.1" |
| tidyverse | "tidyverse" | "2.0.0" |
| timechange | "timechange" | "0.3.0" |
| tinytex | "tinytex" | "0.52" |
| tippy | "tippy" | "0.1.0" |
| tzdb | "tzdb" | "0.4.0" |
| units | "units" | "0.8-5" |
| utf8 | "utf8" | "1.2.4" |
| uuid | "uuid" | "1.2-1" |
| vctrs | "vctrs" | "0.6.5" |
| viridisLite | "viridisLite" | "0.4.2" |
| vroom | "vroom" | "1.6.5" |
| waiter | "waiter" | "0.2.5" |
| waldo | "waldo" | "0.5.3" |
| webshot | "webshot" | "0.5.5" |
| websocket | "websocket" | "1.4.2" |
| withr | "withr" | "3.0.1" |
| wk | "wk" | "0.9.3" |
| xfun | "xfun" | "0.47" |
| xml2 | "xml2" | "1.3.6" |
| xtable | "xtable" | "1.8-4" |
| xts | "xts" | "0.14.0" |
| yaml | "yaml" | "2.3.10" |
| zoo | "zoo" | "1.8-12" |
| base | "base" | "4.2.2" |
| boot | "boot" | "1.3-28" |

| | | |
|---|---|---|
| class | "class" | "7.3-20" |
| cluster | "cluster" | "2.1.4" |
| codetools | "codetools" | "0.2-18" |
| compiler | "compiler" | "4.2.2" |
| datasets | "datasets" | "4.2.2" |
| foreign | "foreign" | "0.8-83" |
| graphics | "graphics" | "4.2.2" |
| grDevices | "grDevices" | "4.2.2" |
| grid | "grid" | "4.2.2" |
| KernSmooth | "KernSmooth" | "2.23-20" |
| lattice | "lattice" | "0.20-45" |
| MASS | "MASS" | "7.3-58.1" |
| Matrix | "Matrix" | "1.5-1" |
| methods | "methods" | "4.2.2" |
| mgcv | "mgcv" | "1.8-41" |
| nlme | "nlme" | "3.1-160" |
| nnet | "nnet" | "7.3-18" |
| parallel | "parallel" | "4.2.2" |
| rpart | "rpart" | "4.1.19" |
| spatial | "spatial" | "7.3-15" |
| splines | "splines" | "4.2.2" |
| stats | "stats" | "4.2.2" |
| stats4 | "stats4" | "4.2.2" |
| survival | "survival" | "3.4-0" |
| tcltk | "tcltk" | "4.2.2" |
| tools | "tools" | "4.2.2" |
| utils | "utils" | "4.2.2" |
